# The nine-year changes of the incidence and characteristics of metabolic syndrome in China: longitudinal comparisons of the two cross-sectional surveys in a newly formed urban community

**DOI:** 10.1186/s12933-016-0402-9

**Published:** 2016-06-03

**Authors:** Boren Jiang, Bin Li, Yongbin Wang, Bing Han, Ningjian Wang, Qin Li, Weihong Yang, Guolan Huang, Jinhua Wang, Yi Chen, Yingchao Chen, Chunfang Zhu, Dongping Lin, Yingli Lu

**Affiliations:** Department of Endocrinology and Metabolism, Shanghai Ninth People’s Hospital, Shanghai Jiaotong University, School of Medicine, Shanghai, 200011 China; Fengcheng Hospital, Fengxian District, Shanghai, China

**Keywords:** Metabolic syndrome, Urbanization, Cross-sectional design, Blood pressure

## Abstract

**Background:**

To assess the 9-year changes of the incidence and characteristics of metabolic syndrome (MetS) in Chinese community under the background of dramatically changed environment.

**Methods:**

Two cross-sectional surveys of the general population were carried out in 2005 and 2014 in Dengmin and Hongnan villages of Fengcheng community, a newly formed urban community from rural area 10 years ago. All permanent adult residents aged 18–80 without active malignant tumors and pregnancy were invited to attend the study. They participated in clinical examinations for anthropometric and blood pressure measurements. Fasting blood samples were drawn for analysis of lipids and glucose. Presence of MetS was defined based on the IDF/AHA harmonized criteria. MetS z-score was calculated to evaluate the degree of total metabolic disorder.

**Results:**

A total of 1042 subjects in 2005 and 1053 subjects in 2014 were included in the final analysis. The participants were stratified by gender. The incidence of MetS was higher in 2014 than 2005 in both genders (female, 48.28 vs 31.61 %; male, 41.12 vs 26.30 %; p value, both <0.001). Of the five MetS components, the FBG and TG levels were higher in 2014 than 2005 in both gender, however, the SBP and DBP values were even lower in 2014 than 2005. The differences of FBG, blood pressure and lipid levels between 2005 and 2014 still exist after ruling out recognized diabetic, hypertensive and dyslipidemic subjects, individually. In MetS subjects, MetS z-score showed the whole metabolic profile get worse in 2014 than 2005 in both sex (female, 1.97 ± 2.53 vs 1.74 ± 2.29; male, 2.51 ± 2.79 vs 1.01 ± 2.38. both P < 0.001). Using 3 abnormal components as a combination, we found the frequency of different kinds of combination also changed in MetS subjects. In female, the combination of WC + BP + HDL disorder decreased from 29.7 % (2005) to 11.0 % (2014) and WC + FBG + BP disorder became the most popular phenotype (18.8 %) in 2014.

**Conclusions:**

The dramatically changed environments have extensive influence on metabolic parameters of local residents. More targeted measures need to be taken to meet the serious challenges of metabolic diseases.

*Trial registration* ChiCTR-ECS-14005052, http://www.chictr.org, Survey on Prevalence in East China for Metabolic Diseases and Risk Factors (SPECT-China)

**Electronic supplementary material:**

The online version of this article (doi:10.1186/s12933-016-0402-9) contains supplementary material, which is available to authorized users.

## Background

Metabolic syndrome (MetS) is becoming a major public health issue [1]. The MetS is a complex of interrelated risk factors for cardiovascular disease and diabetes. These factors include elevated blood pressure (BP), raised fasting blood glucose (FBG), high triglyceride (TG), low high-density lipoprotein cholesterol (HDL-C) levels, and central obesity (waist circumference, WC). With the increasing obesity and sedentary lifestyle during the past decade [2–4], MetS had a rising prevalence worldwide, including China [5–7].

Traditionally, China society was divided into rural–urban dual system according to the economic and cultural differences [8]. People living in urban area have access to more educational and medical resources, more social welfare programs and higher paying jobs than those who live in rural communities [9]. A serial of studies have shown a relatively higher prevalence of MetS in urban area than rural area [10–12], although the opposite trend exists [13]. In order to reduce the imbalance between rural and urban, Chinese government launched the largest-scale urbanization in human history in the past two decades. The process of urbanization was mainly by two ways: the migrants from rural to urban area and in situ urbanization accompanied by urban expansion. The percentage of population living in urban areas was 25.84 % in 1990, 35.39 % in 2000, but the number is 54.7 % by the end of 2014 [14]. This means nearly 29 % Chinese (approximate 350 million) experienced the civilization in the past 20 years. These huge amounts of people experienced remarkably changes of economic and cultural environments in the transition of urbanization. It is pivotal important to know about the influence of urbanization on the incidence and characteristics of metabolic diseases, but data are scarce in China.

Fengcheng community [15] is located in Southern Shanghai, 40 km away from the downtown. This former rural area began urban construction since 2003 and became an urban community in 2009 [16]. The resident living in Fengcheng experienced the rapid shift from peasant to citizen in recent 10 years. In 2005, we carried out a study to screen the incidence of MetS in Fengcheng as a sample of rural area. Nine years later, the MetS incidence was resurveyed in this new urban community. The aim of this study is to investigate the incidence changes of MetS and its components under the background of rapid urbanization and provide evidences for the health provider to take effective actions to tackle the challenges of metabolic diseases.

## Subjects and methods

### Study subjects

#### Design and population

Fengcheng community was regarded as a classical rural agriculture area until 2003. At that time, under the background of fast-going urbanization in China, Fengcheng community began urban construction and the former villager gradually turned into citizen. In 2005, Dengmin, Hongnan and Haigang villages of Fengcheng were selected by cluster random sampling to screening the incidence of MetS in Shanghai suburb [15]. In 2014, we carried out a survey (SPECT-China) to determine the prevalence of metabolic diseases and risk factors in East China (ChiCTR-ECS-14005052, http://www.chictr.org) [17], in which Dengmin and Hongnan villages were chosen again. At that time, the total resident population in Fengcheng community was 9505 (female, 4832). All the eligible permanent adult residents aged 18–80 from each village were invited to attend the study. Individuals who were pregnant or had active malignant tumors were excluded from the study. Of those, 1053 participants agreed and completed the study to give a response rate of 90.2 % (in 2014). The study protocol was approved by the Ethics Committee of Shanghai Ninth People’s Hospital, Shanghai JiaoTong University School of Medicine. All participants provided written informed consent before data collection. For participants who were illiterate, we obtained written informed consent from their proxies.

The two measurements were undertaken by the same group of trained investigators and the biochemical parameters were tested with same method. Subjects were interviewed face-to-face to collect demographic information, medical history of coronary heart diseases, diabetes, hypertension and lipid disorders by using pre-tested questionnaires. The individuals whose income mainly came from field labor were regarded as peasants.

Height and weight were measured to the nearest 0.5 cm and 0.1 kg, respectively, with the participants wearing light-weight clothing and without shoes. Body mass index (BMI) was calculated as weight in kg divided by height in meters. WC was measured on standing participants midway between the lower edge of the costal arch and the upper edge of the iliac crest using a non-elastic tape (to the nearest 0.5 cm). All anthropometric measurements were taken in duplicate and the averages of these measurements were used in the analyses.

Resting systolic and diastolic BP was measured three times at 1-min intervals using a standard mercury sphygmomanometer after a 5-minute rest. The average of the second and the third readings were used in the analyses.

Fasting venous blood samples were collected in the morning after at least 8 h of fasting. The samples for plasma glucose test were collected into vacuum tubes with anticoagulant sodium fluoride and centrifuged on the spot in 1 h after collection. Blood samples were shipped in dry ice to a central laboratory within 2–4 h of collection. FPG, HDL-C and TG were analyzed enzymatically using an autoanalyzer. All laboratory equipment was calibrated, and blinded duplicate samples were used for these analyses.

#### Definition of metabolic syndrome

MetS was defined based on the IDF/AHA harmonized criteria [17]. Thus, positive diagnosis of the syndrome was established when at least three of the following were present: (1) Waist circumference ≥90 cm in men or ≥80 cm in women [18]; (2) HDL cholesterol <1.0 mmol/L in men or <1.3 mmol/L in women; (3) Serum TGs ≥1.7 mmol/L (4) Serum glucose level ≥5.6 mmol/L; (5) Blood pressure ≥130/85 mmHg. Treatment with anti-hypertensive, hypoglycemic or lipid-lowering drugs was considered as alternate indicators of the latter three components. To compare the incidence difference between 2005 and 2014, age-standardized incidence rates of MetS were calculated using direct standardization with population composition of the Sixth National Population Census of China (2010). In order to exclude the influence of medication or other treatment measures after the diagnosis of metabolic diseases, we sorted out the subjects with or without recognized Diabetes Mellitus (DM), hypertension and dyslipidemia history for subgroup analysis. The 1999 World Health Organization (WHO) diagnostic criteria were used to diagnose diabetes mellitus [19]. A subject with SBP or DBP ≥140/90 mmHg is considered hypertension by WHO/ISH 1999 criteria [20].

MetS z-score was calculated. It takes into account continuous changes in each component, representing the score of continuous risk for MetS [21]. For each risk factor, a z-score was calculated (individual value—sample mean/standard deviation of the sample). For the blood pressure, we used the MAP (2/3 DBP + 1/3 SBP) of for calculating the score. Total score = waist Z score + BP Z score + glucose Z score + HDL-C Z score + triglycerides Z score. A lower risk score is indicative of a better metabolic profile.

### Statistical analysis

All analyses were stratified by sex. Continuous variables are presented as mean ± SD and proportions were calculated for discrete variables. To test differences in continuous variables between 2005 and 2014, the independent sample t test was performed. Associations between nominal variables were performed with the Pearson Chi square test. Since the mean age of study participants was higher in 2014 than 2005, age-adjusted linear and logistic regression models were used to compare means and proportions of different components of the MetS. General linear model were used to evaluate whether the difference was maintained after controlling for potential confounding variables. We normalized FBG by inverse transformation and TG by a logarithmic transformation before evaluated in *t* test or general linear model. Paired t tests were performed to determine any significant changes in population attending both surveys. In the process of analyzing the frequency of components combination of MetS, all kinds of 3-factor combinations were counted. The actual frequency of one 3-factor combination is equal to the sum of the number of this combination in 3-factor group and the number of 4-factor and 5-factor group including this 3-factor combination [The theoretical frequency of total random 3 parameters combination equal to (total 3-factor frequency)*1 plus (total 4-factor frequency)*4 plus (total 5-factor frequency)*5]. Data were analyzed using SPSS software version 22.0 for Mac, with the significance level set at p < 0.05 for all analyses.

## Results

A total of 1042 subjects (females, 658) with a mean ± SD age of 53.72 ± 12.14 years in 2005 and 1053 subjects (females, 642) with age 57.08 ± 12.46 years old in 2014 were included in the study. All participants were of Han origin. From Table 1, the residents still doing agricultural work were markedly reduced in 2014 than 2005. Compare with 2005, the percentage of recognized coronary heart disease, type 2 DM, hypertension and dyslipidemia were much higher in 2014.

**Table 1:** General characteristics of the study population in 2005 and 2014

	2005 (n = 1042)	2014 (n = 1053)	P value
Gender (female)	658 (63.15)	642 (60.97)	NS
Age (years)	53.72 ± 12.14	57.08 ± 12.46	<0.001
Occupation (peasants)	587 (56.33)	254 (24.12)	<0.001
BMI (kg/m^2^)	24.18 ± 3.28	25.26 ± 3.53	<0.001
MetS	309 (29.65)	479 (45.49)	<0.001
Recognized diseases			
CHD	34 (3.26)	116 (11.02)	<0.001
Type 2 DM	33 (3.17)	92 (8.74)	<0.001
Hypertension	233 (22.36)	381 (36.18)	<0.001
Dyslipidemia	32 (3.07)	100 (9.50)	<0.001

Data were showed as mean ± SD or number of subjects (%)

*BMI* body mass index, *CHD* coronary heart disease, *DM* diabetes mellitus

In view of different incidence and variable cut-points between female and male, we divided the subjects by gender in the following analysis. Using the IDF/AHA harmonized criteria, the MetS incidence was significantly elevated in 2014 than 2005 (female, 48.28 vs 31.61 %; male, 41.12 vs 26.30 %; p value, both <0.001) and these differences still exist after accounting for age and BMI. In terms of components of MetS, the FBG and TG levels were higher in 2014 than 2005 in both sex, however, the SBP and DBP values were even lower in 2014 than 2005. These differences still exist after adjusting age and BMI. WC had a tendency of increment in 2014 but cannot reach statistical significance. The HDL levels were higher in 2014 than 2005 in female, but the difference was absent in male. The percentage of individual component that reached the MetS criteria showed similar trend with the continuous variables in both genders (Table 2).

**Table 2:** Comparison of MetS components between 2005 and 2014

	Female		P value	Male		P value
	2005 (n = 658)	2014 (n = 642)		2005 (n = 384)	2014 (n = 411)	
WC (cm)	80.38 ± 8.69	80.89 ± 10.07	0.33	83.91 ± 9.01	84.69 ± 9.64	0.24
FBG (mmol/L)	4.99 ± 1.19	5.88 ± 1.47	<0.001	5.24 ± 1.36	5.99 ± 1.45	<0.001
SBP (mmHg)	139.91 ± 24.61	134.13 ± 22.42	<0.001	143.24 ± 23.56	135.14 ± 19.76	<0.001
DBP (mmHg)	86.45 ± 12.50	79.83 ± 13.69	<0.001	89.92 ± 12.94	80.35 ± 13.23	<0.001
TG (mmol/L)	1.20 ± 0.89	1.56 ± 0.96	<0.001	1.24 ± 0.94	1.80 ± 1.54	<0.001
HDL (mmol/L)	1.38 ± 0.30	1.49 ± 0.32	<0.001	1.34 ± 0.34	1.36 ± 0.31	0.35
Numbers of each component reach MetS criteria (%)						
WC	329 (50)	468 (72.90)	<0.001	110 (48.18)	140 (49.88)	0.63
FBG	94 (14.29)	302 (47.04)	<0.001	113 (29.42)	214 (52.06)	<0.001
BP	460 (69.91)	421 (65.58)	0.095	304 (79.17)	289 (70.32)	0.004
TG	112 (17.02)	192 (29.91)	<0.001	75 (19.53)	143 (34.79)	<0.001
HDL	291 (44.22)	192 (29.91)	<0.001	47 (12.23)	36 (8.76)	0.11
MetS incidence rate (%)						
Crude	31.61	48.28	<0.001	26.30	41.12	<0.001
ASR	24.47	36.90	<0.001	25.26	36.49	<0.001

Data were showed as mean ± SD or number of subjects (%). *ASR* age-standardized incidence rate (using China standard population 2010). Data were showed as number of subjects (%) or mean ± SD

**Fig. 1:**
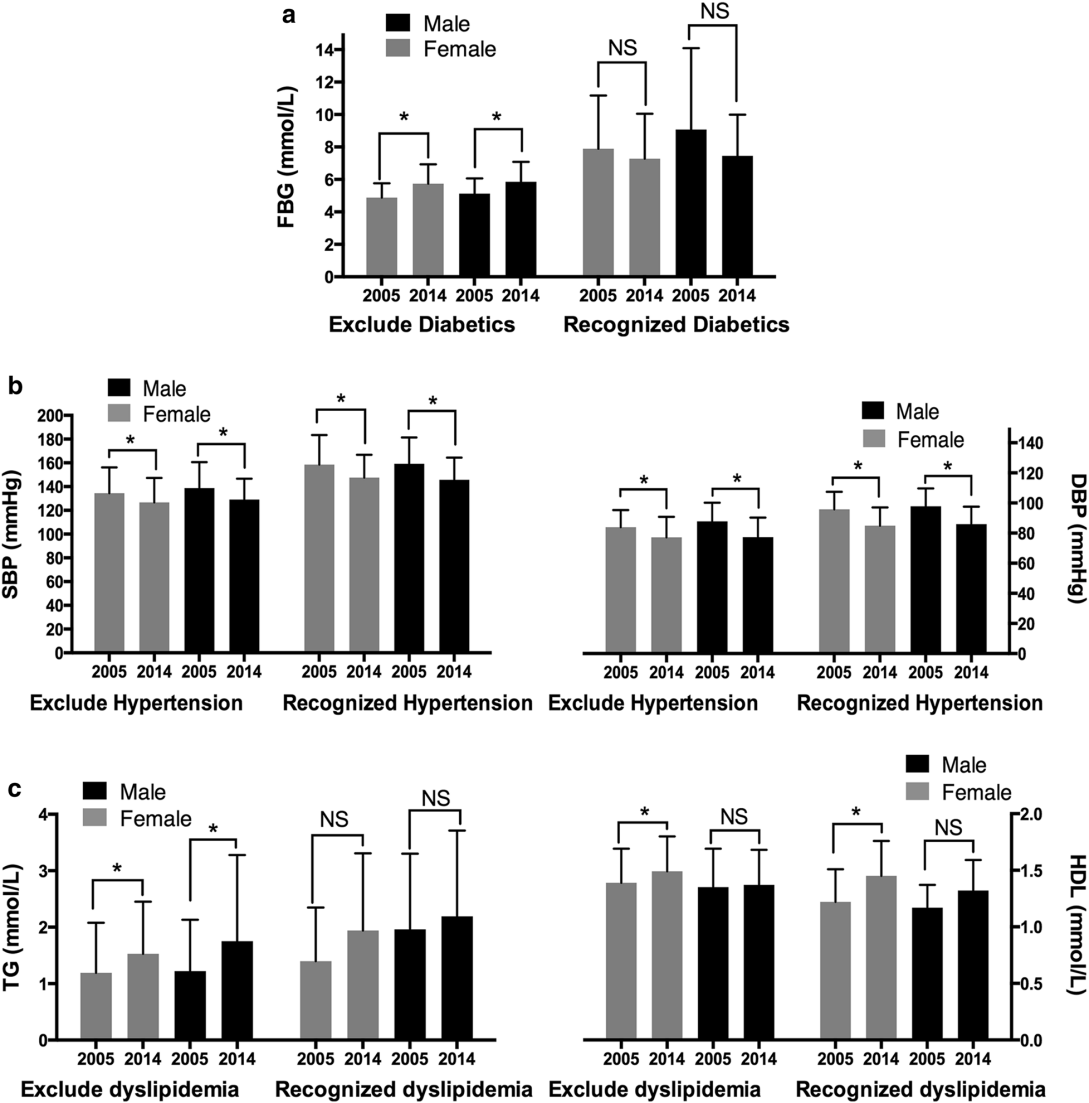
Comparisons of FBG (**a**), blood pressure (**b**) and lipid levels (**c**) between 2005 and 2014 stratified by recognized type 2 DM, hypertension and dyslipidemia, individually. *P < 0.05; *NS* not significant

We sorted out the subjects with or without recognized DM, hypertension and dyslipidemia history for further analysis. From Fig. 1, the FBG, blood pressure and lipid levels differences between 2005 and 2014 after ruling out recognized type 2 DM, hypertension and dyslipidemia, individually, showed similar results with the total subjects in both genders. In patients with recognized type 2 DM, hypertension and dyslipidemia, the SBP and DBP levels in 2014 were still lower than 2005 in hypertensive individuals in both genders (P < 0.05). HDL levels were higher in 2014 in female patients with dyslipidemia history and the male showed the same trend (P < 0.05). The FBG levels had a tendency of decrease in diabetic patients in both genders.

In view of the opposite alteration of BP and BG, TG and HDL on MetS incidence, we further analyzed the change of metabolic disorder spectrum in the MetS population. From Table 3, the frequency of metabolic disorder in male followed the same pattern (BP > WC > FBG > TG > HDL), but the frequency of HDL abnormality was lower and glucose showed marginal higher in 2014 than 2005. In female, because of the markedly increased rate of abnormal FBG and TG, together with decreased rate of abnormal BP and HDL, the frequency of metabolic disorder changed from (BP > WC > HDL > TG > FBG) to (WC > BP > FBG > TG > HDL). To quantify the total impact of metabolic disorder, we calculated the MetS z-score and found despite of the frequency change of different components, the whole metabolic profile get worse in 2014 than 2005 in both sexes (female, 1.97 ± 2.53 vs 1.74 ± 2.29; male, 2.51 ± 2.79 vs 1.01 ± 2.38. both P < 0.001), these differences still exist after adjusting age.

**Table 3:** The frequency of abnormal components in MetS subjects between 2005 and 2014

	Female MetS		P value	Male MetS		P value
	2005 (n = 208)	2014 (n = 310)		2005 (n = 101)	2014 (n = 169)	
Abnormal component						
WC	191 (91.83)	288 (92.90)	0.649	88 (87.13)	143 (84.62)	0.57
FBG	73 (35.10)	234 (75.48)	<0.001	66 (65.35)	129 (76.33)	0.051
BP	202 (97.12)	269 (86.77)	<0.001	98 (97.03)	158 (93.49)	0.204
TG	75 (36.06)	170 (54.84)	<0.001	54 (53.47)	107 (63.31)	0.11
HDL	157 (75.48)	150 (48.39)	<0.001	32 (31.68)	30 (17.75)	0.008
MetS z score	1.74 ± 2.29	1.97 ± 2.53	<0.001	1.01 ± 2.38	2.51 ± 2.79	<0.001

Data were showed as number of subjects (%) or means ± SD

**Fig. 2:**
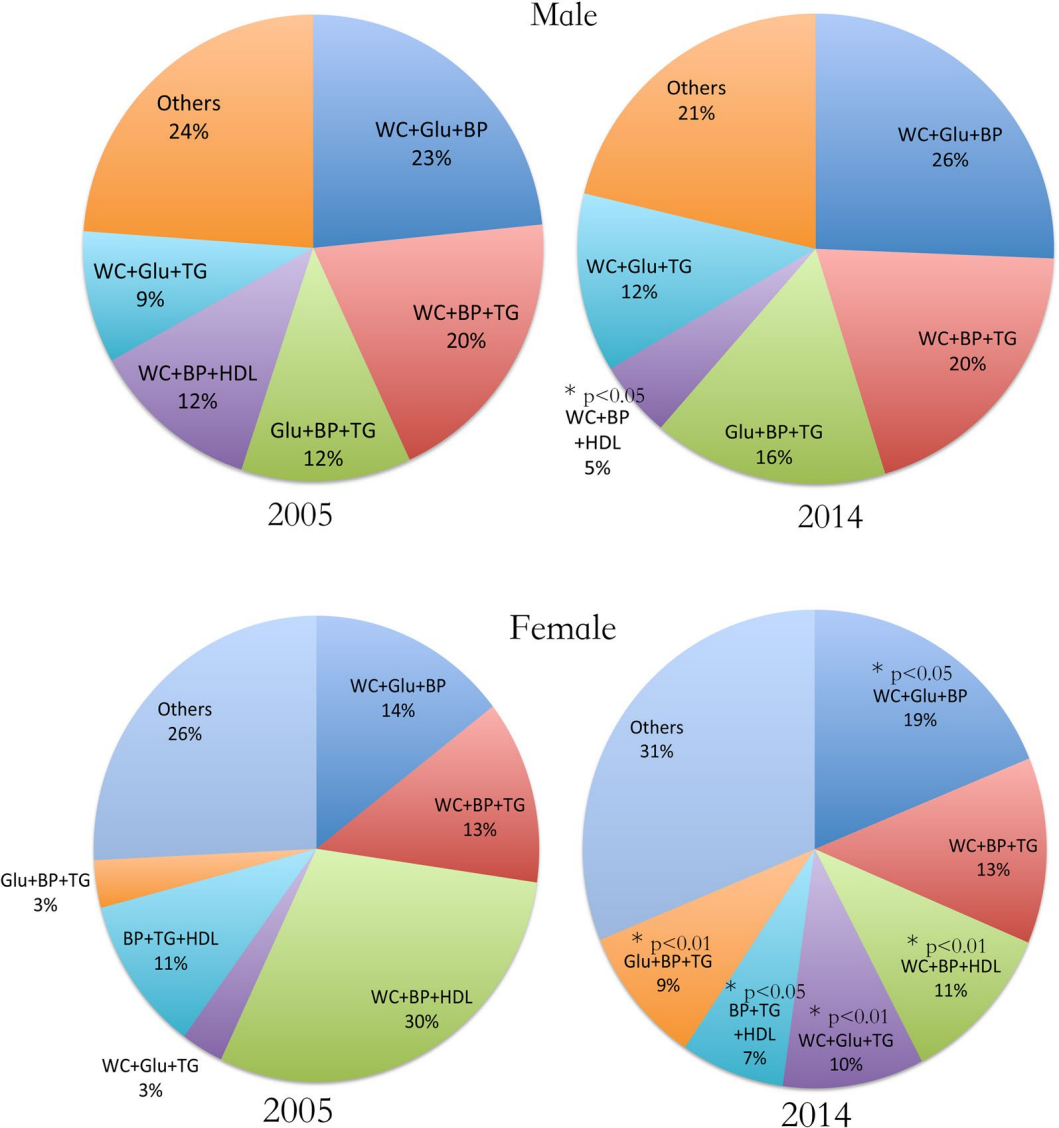
Comparison of various components clustering in all MetS subjects between 2005 and 2014

The clustering of various components was further assessed. Additional file 1: Table S1 illustrated metabolic disorder patterns in all MetS subjects. Using 3 parameters as a combination, we found the combinations of components were similar between 2005 and 2014 in male, except the WC + BP + HDL was slightly decreased in 2014. However, in female, the phenotype of WC + BP + HDL markedly decreased from 29.7 % (2005) to 11.0 % (2014). WC + FBG + BP became the most frequent combination (18.8 %) in 2014 (Fig. 2).

In 2014, there were 594 residents attended the first survey (2005). After 9 years, BMI increased significantly in both sex but WC only showed slightly rise. FBG, TG and HDL concentrations were higher than before except the HDL in male showed no difference. SBP and DBP levels had a tendency to decline except SBP in female (Table 4).

**Table 4:** Characteristics of the repeatedly surveyed population in 2005 and 2014

	Female (n = 396)		P value	Male (n = 198)		P value
	2005	2014		2005	2014	
Age (years)	52.30 ± 10.07	61.30 ± 10.07	<0.001	53.80 ± 8.98	62.80 ± 8.98	<0.001
BMI (kg/m^2^)	24.31 ± 3.28	25.46 ± 3.48	<0.001	24.17 ± 3.03	25.59 ± 3.33	<0.001
WC (cm)	81.30 ± 8.47	81.97 ± 9.63	0.171	83.63 ± 8.54	85.61 ± 9.57	0.002
FBG (mmol/L)	4.93 ± 0.89	6.00 ± 1.45	<0.001	5.21 ± 1.24	6.06 ± 1.48	<0.001
SBP (mmHg)	139.17 ± 22.45	136.67 ± 22.07	0.057	145 ± 23.32	137.42 ± 20.70	<0.001
DBP (mmHg)	87.00 ± 12.90	81.64 ± 14.13	<0.001	91.64 ± 12.82	81.47 ± 13.76	<0.001
TG (mmol/L)	1.21 ± 0.83	1.61 ± 0.96	<0.001	1.21 ± 1.00	1.72 ± 1.44	<0.001
HDL (mmol/L)	1.35 ± 0.28	1.50 ± 0.33	<0.001	1.34 ± 0.35	1.36 ± 0.31	0.414
MetS n (%)	142 (35.9)	193 (48.7)	<0.001	52 (26.3)	84 (42.2)	<0.001

Data were showed as mean ± SD or number of subjects (%)

## Discussion

In this study, the changes of metabolic syndrome incidence and its components were explored in a typical community before and after urbanization. It is not surprising that the incidence of MetS is higher over a period of 9 years in the same community. The key finding of our study is the higher incidence of MetS was mainly attributed to more FBG and TG abnormality, but not WC, BP and HDL. The blood pressure and HDL disorders even showed a beneficial change. Eventually, the risk factor combination also altered dramatically. These unique alterations provide first-hand evidence for the health provider to take corresponding actions.

In our study, the incidence of MetS in 2014 was much higher than 9 years before. Using MetS z-score, we also found the whole metabolic profile got worse in 2014 than 2005 in MetS subjects. In most developing countries, the prevalence of MetS is still increasing [6, 22, 23]. Fortunately, in some developed area, such as France, had showed trend of decline [24]. US also experienced an increase from 1990s to 2000s [5] and decreased tendency from 25.5 % to 22.9 % (1999/2000 to 2009/2010) [25]. Further follow-up is needed in our community to observe the possible shift.

One interesting finding of our research is the increased incidence of MetS was mainly attributed to more FBG and TG disorders. However, the WC did not change significantly in both genders. This is different from other developing area, in which the increasing prevalence of obesity was still regarded as the pivotal cause of MetS [22, 26]. Even in US, the prevalence of elevated waist circumference increased from 45.4 to 56.1 % (1999/2000 to 2009/2010) [25]. By contrast, the above-mentioned France study showed prevalence of abdominal obesity was unchanged (from 1996 to 2006) [24]. In addition, in our community the BMI increased significantly in 2014 compared with 2005, suggesting the increased body weight were stored in non-abdominal area. We cannot explain our WC data clearly till now and further measurement of body fat distribution will be helpful to explain current findings [27]. More FBG and TG disorders are similar to other developing area. Excess sugar and lipid-rich food intake (classic western food) under urbanization may be the underlying cause. However in developed country, the hypertriglyceridemia [25] and elevated fasting glucose [24] prevalence showed decreasing trends.

The good news for us is the BP and HDL disorders decreased significantly (except HDL in male). To our knowledge, increased BP was reported almost all large-scale clinical trials in china. But in most of developed area, including Japan [22] and US, BP had showed tendency of decline. Some articles argued this decline was related to the antihypertensive medicine. From our data, the SBP and DBP levels in 2014 were still lower than 2005 after ruling out the cases with hypertension history. Although we cannot make sure whether our community had successfully passed the blood pressure plateau, the decrease of BP per se is an exciting result. The reason we think could be partly attributed to the lower salt intake after life style education. As to higher HDL, Framingham Heart Study found an increase in HDL-C during a 10-year period (from 1991 to 2001) [28]. One study in Nigeria showed the HDL-C levels was higher among urban dwellers than rural dwellers [29]. The NHANES analysis illustrated a slight increase of age-adjusted HDL-C in women (from 53.8 mg/dL in 1976–1980 to 55.9 mg/dL in 1999–2002), but not in men. The NHANES data also found a borderline significant increase in TG levels when restricting their sample to adults aged 20 to 74 years [30]. These data are quite similar to us. There is evidence that the increase in HDL-C and the decrease in TG are due to changes in cigarette smoking [31] and carbohydrate intake [32]. The exact cause of beneficial HDL change in our rapid urbanized community needs further investigation.

Another good news is the higher awareness of metabolic disease and better control of hypertension and diabetes in 2014 than 2005. We think the reason is more extensive medical insurance coverage, more hospital and physician service accessibility after the urbanization [33].

The major threat of MetS is its association with increased cardiovascular (CV) events and all-cause mortality rates [34]. CV diseases are among the top health problems of the Chinese people, and pose a serious challenge to the public health system [35, 36]. However, the relative contribution of the 5 risk factors to the occurrence of CV diseases is still unknown. One follow-up study illustrated that patient with diabetes suffered from significantly increased mortality rate after CABG (coronary artery bypass grafting), but the subjects with MetS showed no difference with the matched background population [37]. Hypertriglyceridemia is associated with a substantially increased long-term total mortality and CV risk [38], which is regarded as a potential CV residual risk factor [39]. In our community, more glucose and TG disorders occurred after 9-year observation. The effects of these changes, together with beneficial shift of BP and HDL disorders, on the occurrence of CV diseases need further follow-up in our community.

Another issue we need to address is the possible involvement of non-alcoholic fatty liver disease (NAFLD). Several studies have independently provided evidence for a strong association between NAFLD and each component of the metabolic syndrome, including central obesity, hyperglycemia, dyslipidemia, and hypertension [40]. The growing glucose and lipid disorders in our community may be related to NAFLD.

Although certain components of MetS showed beneficial changes, but the total metabolic profile got worse in 2014 than 2005. That means the risk of MetS complications was still higher in 2014. In the components combination analysis, we found the combination pattern changed markedly in female and more female MetS subjects had got total five risk factors. It provides important information for us to take more specific actions for this group.

Intensive lifestyle intervention or specific nutrient substitution has been reported to produce benefit in multiple metabolic cardiovascular risk factors [41]. In our community, targeted measures to reduce the TG and FBG levels will be the key point to control the rapid increase of MetS.

Some limitations need to be addressed in our study. First, choosing a rural area without experiencing urbanization as control group will be quite helpful to our interpretation. Unfortunately, we did not have that opportunity. Second, as we mentioned in the introduction, urbanization includes the migrants from rural to urban area and in situ urbanization accompanied by urban expansion. A recently published cross-sectional study had showed differences of metabolic risk factors between internal migrant workers and general population [42]. Our sample was from in situ urbanization and the MetS characteristics of migrants still need further investigation. Third, large regional gap exists between big cities (like Shanghai) and other medium or small city, thus there must be economic or medical differences after the urbanization in different areas. Data from Guangdong province in southern China showed similar results on prevalence of hyperglycemia and TG from 2002 to 2010 in rural area, but they did not found the decreased trend of BP and HDL [43]. It is important to broaden the sample size to get more accurate and generalizable results. Fourth, other confounders, such as different nationalities, socioeconomic position, service availability and residential density, noise pollution, drugs, diets, physical activity may also influence the results [44].

In summary, our study present a unique scenario of the characteristics of MetS under the rapid urbanization in China, but the reasons underlying are complex. On the one hand, the higher incidence and more FBG and TG disorders still needed more efforts to deal with, on the other, the beneficial changes of blood pressure and HDL and the more awared and better controlled diabetes and hypertension mean brilliant prospects of urbanization to rural Chinese people. The local government needs to take corresponding measures to help the old residents adjust the new urbanized environment.

## Conclusions

The dramatically changed environments have extensive influence on metabolic parameters in the inhabitant Chinese people. More targeted measures need to be taken to meet the serious challenges of metabolic diseases.

## Additional file

10.1186/s12933-016-0402-9 The clustering of various components in MetS subjects.

## Abbreviations

BMI: body mass index
BP: blood pressure
CHD: coronary heart disease
DM: diabetes mellitus
FBG: fasting blood glucose
HDL-C: high-density lipoprotein cholesterol
MetS: metabolic syndrome
TG: high triglyceride
WC: waist circumference
