# Supplementary material for: The nine-year changes of the incidence and characteristics of metabolic syndrome in China: longitudinal comparisons of the two cross-sectional surveys in a newly formed urban community
Source: Cardiovasc Diabetol. 2016 Jun 3;15:84. doi: 10.1186/s12933-016-0402-9 (PMC4891912; doi:10.1186/s12933-016-0402-9)
Supplement: Supplementary file 1 — 10.1186/s12933-016-0402-9 The clustering of various components in MetS subjects. [file 12933_2016_402_MOESM1_ESM.docx]

|  | Female MetS | |  | Male MetS | |  |
| --- | --- | --- | --- | --- | --- | --- |
|  | 2005(n=208) | 2014(n=310) | p value | 2005(n=101) | 2014(n=169) | p value |
| 3 factors | 142 (68.27) | 176(56.77) | <0.05 | 70(69.31) | 120(71.01) | NS |
| WC+FBG+BP | 29 (13.94) | 85(27.42) | <0.01 | 27(26.73) | 56(33.14) | NS |
| WC+FBG+TG | 0 | 10(3.23) | <0.01 | 2(1.98) | 5(2.96) | NS |
| WC+FBG+HDL | 2(0.96) | 8(2.58) | NS | 1(0.99) | 0 | NS |
| WC+BP+TG | 15(7.2) | 26(8.39) | NS | 18(17.82) | 29(17.16) | NS |
| WC+BP+HDL | 75(36.06) | 23(7.42) | <0.01 | 9(8.91) | 3(1.78) | <0.05 |
| WC+TG+HDL | 4(1.92) | 9(2.90) | NS | 0 | 2(1.18) | NS |
| FBG+BP+TG | 1(0.48) | 5(1.61) | NS | 8(7.92) | 19(11.24) | NS |
| FBG+BP+HDL | 4(1.92) | 3(0.97) | NS | 4(3.96) | 2(1.18) | NS |
| FBG+TG+HDL | 0 | 7(2.26) | <0.05 | 0 | 3(1.78) | NS |
| BP+TG+HDL | 12(5.77) | 0 | <0.01 | 1(0.99) | 1(0.06) | NS |
|  |  |  |  |  |  |  |
| 4 factors | 58(27.88) | 87(28.06) | NS | 27(26.73) | 38(22.49) | NS |
| WC+FBG+BP+TG | 6(2.88) | 34(10.97) | <0.01 | 14(13.86) | 30(17.75) | NS |
| WC+FBG+BP+HDL | 23(11.06) | 21(6.77) | NS | 6(5.94) | 1(0.06) | <0.05 |
| WC+BP+TG+HDL | 29(13.94) | 18(5.81) | <0.01 | 7(6.93) | 5(2.96) | NS |
| WC+FBG+TG+HDL | 0 | 7(2.26) | <0.05 | 0 | 1(0.06) | NS |
| FBG+BP+TG+HDL | 0 | 7(2.26) | <0.05 | 0 | 1(0.06) | NS |
|  |  |  |  |  |  |  |
| 5 factors | 8(3.85) | 47(15.16) | <0.01 | 4(3.96) | 11 (6.51) | NS |

Supplementary table S1: The clustering of various components in MetS subjects.

Data were showed as number of subjects (%).
